# Improving Domain Wall Thermal Switching and Dynamics in Perpendicular Magnetic Anisotropy Nanowire for Reliable Spintronic Memory

**DOI:** 10.3390/nano15201552

**Published:** 2025-10-11

**Authors:** Mohammed Al Bahri, Salim Al-Kamiyani

**Affiliations:** Department of Basic and Applied Sciences, A’Sharqiyah University, P.O. Box 42, Ibra 400, Oman

**Keywords:** perpendicular magnetic anisotropy, domain wall, thermal switching, micromagnetic simulation, nanowire geometry

## Abstract

The random thermal switching of domain walls (DWs) in perpendicularly magnetized anisotropy nanowires (PMA) poses a significant challenge for the reliability of spintronic storage devices. In this work, we study the thermal nucleation and dynamics of DWs in PMA nanowires using micromagnetic simulations. The focus is on the effect of device temperature, with attention to uniaxial anisotropy energy (*K_u_*), saturation magnetization (*M_s_*), and nanowire geometry. The results show that larger *K_u_* or *M_s_* reduces DW thermal switching, thereby enhancing DW thermal stability and increasing the DW nucleation temperature (*T_n_*). A wider or thicker nanowire also lowers the probability of thermally induced DW creation, further improving stability. In addition, DW velocity rises with temperature, showing a thermally assisted motion. These results provide useful guidance for designing PMA-based memory devices with improved resistance to thermal fluctuations.

## 1. Introduction

The emergence of spintronic technologies has spurred significant interest in utilizing domain walls (DWs) in magnetic nanowires as active elements for memory and logic devices due to their non-volatility, high speed, and energy efficiency [1,2,3]. Among the various magnetic configurations, perpendicularly magnetized anisotropy nanowires (PMA) have attracted considerable attention for next-generation spintronic applications, including racetrack memory and magnetic random-access memory (MRAM) [4,5,6,7,8]. PMA systems offer enhanced data retention and thermal stability compared to in-plane systems, primarily due to the larger uniaxial anisotropy energy that maintains out-of-plane magnetization alignment [9,10,11]. This intrinsic robustness positions PMA structures as promising candidates for high-density, thermally reliable storage media. Despite the advantages, one critical challenge remains: the thermal activation of domain walls under operating conditions. Thermal fluctuations at the nanoscale can induce stochastic DW nucleation, motion, or annihilation, which compromises device reliability and performance [12,13,14]. Understanding how thermal energy interacts with key magnetic parameters, namely the uniaxial anisotropy constant (*K_u_*), saturation magnetization (*M_s_*), and nanowire geometry, is essential for mitigating thermal instabilities [15,16,17,18,19]. Prior studies have demonstrated that DW motion can be thermally assisted [20,21], and that magnetic properties like *K_u_* and *M_s_* significantly influence the thermal energy required for DW nucleation [22,23]. Although the qualitative influence of anisotropy, saturation magnetization, and nanowire geometry on DW thermal stability is well established, there is still a lack of systematic and quantitative comparison of their collective effects under current-driven (STT) and thermally fluctuating conditions in PMA nanowires. Several experimental and computational investigations have reported that increasing *K_u_* enhances thermal stability by raising the energy barrier for DW nucleation [24,25]. Similarly, a higher *M_s_* contributes to stronger magnetic ordering, further resisting thermal agitation [26,27]. From a geometric perspective, increasing the width and thickness of a nanowire increases the magnetic volume, which in turn elevates the total anisotropy energy, resulting in more thermally stable structures [28,29,30]. These findings are supported by micromagnetic simulations and experimental studies on multilayered thin films and nanowires [31,32,33,34,35]. Current-induced effects, particularly spin-transfer torque (STT), add another layer of complexity to DW dynamics in PMA systems [36,37,38,39]. While higher current densities can facilitate faster DW motion and switching, they may also reduce the DW nucleation temperature by aiding in thermal destabilization of the magnetic configuration [40,41]. Hence, it is essential to optimize both magnetic and geometric parameters while considering the role of STT to achieve a reliable trade-off between fast switching and thermal robustness. Thermal stability becomes even more crucial as device dimensions shrink. The energy barrier against spontaneous DW nucleation scales with the volume (V) and *K_u_*, making ultrathin or narrow wires particularly susceptible to thermal noise [42,43,44,45]. Therefore, engineering domain wall thermal stability through systematic tuning of magnetic material properties and nanowire geometry is essential for ensuring data integrity in practical devices. The novelty of this work lies in its systematic and quantitative comparison of the combined influence of intrinsic parameters (*K_u_*, *M_s_*) and extrinsic factors (geometry, temperature) under the dual effects of spin-transfer torque and thermal activation. Using micromagnetic simulations, we show how these parameters collectively determine DW nucleation temperature, thermal stability, and velocity evolution across creep and flow regimes [18]. The analysis reveals that increasing either *K_u_* or *M_s_* raises the DW nucleation temperature, significantly boosting thermal stability. Moreover, increasing nanowire width or thickness also enhances robustness by enlarging the magnetic volume and associated anisotropy energy. In parallel, DW velocity evolution is shown to increase with temperature, transitioning through creep and flow regimes in a manner consistent with experimentally measured trends.

By quantifying these dependencies, this work systematically combines intrinsic (*K_u_*, *M_s_*) and extrinsic (geometry, temperature) factors to provide practical insights into strategies for optimizing reliable spintronic memory devices. Importantly, we explore a broad temperature window (0–600 K) and identify the domain wall velocity saturation above 550 K, linking it to thermal fluctuations near the Curie temperature. These results provide a comprehensive perspective on the collective influence of magnetic and geometric factors on DW dynamics and offer practical insights for the design of high-density, robust spintronic memory devices capable of operating reliably under thermal stress.

## 2. Theoretical Model

**Figure 1:**
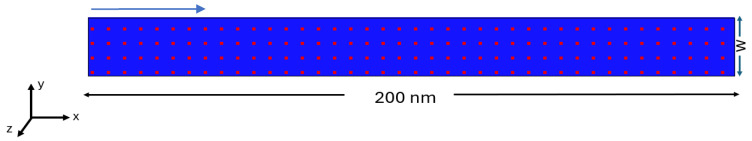
Schematic representation of the perpendicularly magnetized nanowire geometry used in the micromagnetic simulations. The planar nanowire has a fixed length of 200 nm, a width of 40 nm, and a thickness of 5 nm. an applied current density of J = 1.11 × 10^11^ A/m^2^ and T = 0 K.

Micromagnetic simulations were conducted using the Object Oriented MicroMagnetic Framework (OOMMF) software (version is 1.2a5) [45] on nanowires with lengths of 200 nm, widths ranging from 30 nm to 60 nm, and thicknesses between 3 nm and 9 nm, with as illustrated in Figure 1. The simulation device was discretized into small computational cells with dimensions of 2.5 nm × 2.5 nm × 5 nm, ensuring that each cell size remains smaller than the material’s exchange length, which is given by and calculated to be 5.3 nm. The uniaxial anisotropy axis was aligned along the z-direction to represent PMA. To ensure clarity, the parameters used in this work were consistently defined. The nanowire length was fixed at 200 nm, while the width (30–60 nm) and thickness (3–9 nm) were systematically varied. The exchange stiffness was kept constant at A = 1.3 × 10^−11^ J/m, with Gilbert damping α = 0.01 and the non-adiabatic parameter β = 0.04. The saturation magnetization *M_s_* was varied between 700 and 800 kA/m to represent realistic thermal conditions, and the anisotropy constant *K_u_* was adjusted between 0.1 and 1 × 10^5^ J/m^3^. In all simulations, the saturation magnetization (*M_s_*) and the uniaxial anisotropy constant (*K_u_*) were kept fixed and not varied as functions of temperature. Current densities in the range of 3.13–5.21 × 10^11^ A/m^2^ were applied, and the simulations were conducted over a temperature window of 0–600 K. These ranges were chosen to reflect experimentally relevant values typically reported for multilayer PMA systems such as [Co/Pt] and [CoFeB/MgO] stacks, ensuring that the findings are representative of realistic device conditions. The simulations incorporated both spin-transfer torque (STT) and thermal fields using the Landau–Lifshitz–Gilbert equation extended with temperature-dependent fluctuations.

(1) $$\frac{dm}{dt}=-\gamma m\times H_{eff}+\alpha m\times \frac{dm}{dt}-\left(u.\nabla \right)m+\beta m\times \left(u.\nabla \right)m$$

where m is the normalized magnetization vector, γ is the gyromagnetic ratio, α is the Gilbert damping constant, $H_{eff}$ is the effective magnetic field (including anisotropy, exchange, demagnetizing, and thermal fields), u is the effective drift velocity associated with the spin-polarized current, defined as $u=(gP\mu_{b}/2eM_{s})J$, β is the non-adiabatic STT parameter [18,46].

Initial magnetization was uniformly set in the negative z-direction, and current was applied along the wire axis from left end.

Domain wall velocity was obtained by tracking the temporal evolution of the domain wall center position along the nanowire. The wall position was determined from the zero-crossing of the spatially averaged out-of-plane magnetization component (m_z_) across the wire. The instantaneous velocity was calculated as the slope of the wall displacement versus time curve, and average velocities were extracted once steady motion was established. This procedure is consistent with common micromagnetic analysis of current-driven DW motion in OOMMF.

## 3. Results and Discussion

**Figure 2:**
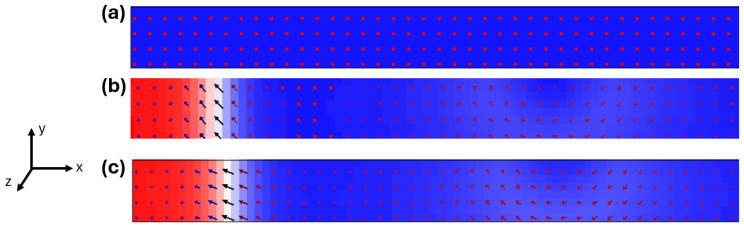
Micromagnetic snapshots showing magnetization states in a PMA nanowire (200 × 40 × 10 nm^3^) at different temperatures and J = 5.21 × 10^11^ A/m^2^: (**a**) initial magnetization at 0 K with uniform out-of-plane alignment, (**b**) domain wall nucleation initiated at 100 K, and (**c**) a well-defined domain wall formed and propagated at 300 K. Arrows indicate the local magnetization direction, and color contrast represents the out-of-plane magnetization component.

This study initially investigates how variations in device temperature influence the orientation of magnetization in perpendicularly magnetized nanowires. Figure 2 illustrates the evolution of domain wall (DW) nucleation in a perpendicularly magnetized nanowire as a function of temperature, providing a visual understanding of thermally induced transitions in magnetization states, specifically the nucleation and evolution of domain walls in perpendicularly magnetized nanowires. At 0 K [Figure 2a], the magnetization remains uniformly aligned in the negative z-direction, indicating a fully saturated and stable magnetic state with no thermal excitations. This scenario represents the ideal ground state of the PMA nanowire, where the high anisotropy energy and absence of thermal agitation prevent any form of magnetic reversal or domain wall formation. As the temperature increases to 100 K [Figure 2b], thermal energy is sufficient to nucleate a domain wall. The uniform magnetic alignment near the edges of the wire breaks down, leading to localized magnetization reversal and the formation of a well-defined wall. This early nucleation highlights the system’s sensitivity to thermal activation, even at relatively low temperatures. The wall tends to form preferentially near the boundaries, where demagnetizing effects lower the energy barrier compared to the wire interior. By 300 K [Figure 2c], a clearly defined domain wall forms and extends across the width of the nanowire. The domain wall becomes more pronounced and stable due to the increased thermal energy overcoming the anisotropy barrier in localized regions, allowing the magnetization to reverse and form a distinct wall separating regions of opposite magnetization. This observation demonstrates that thermal energy not only initiates DW nucleation but also facilitates its propagation, especially in geometries where spin-transfer torque or other external stimuli are not dominant. The sequence from 0 K to 300 K highlights the threshold-like behavior of thermally activated DW dynamics in PMA systems. It underscores the importance of thermal fluctuations as a fundamental driving force in domain wall nucleation and stability. These results have strong implications for spintronic device engineering, especially in applications such as racetrack memory, where thermal noise can either enhance switching or degrade stability, depending on design parameters. By controlling key factors like anisotropy, nanowire geometry, and operational temperature, one can modulate the onset and robustness of domain wall formation to achieve reliable data storage and processing.

**Figure 3:**
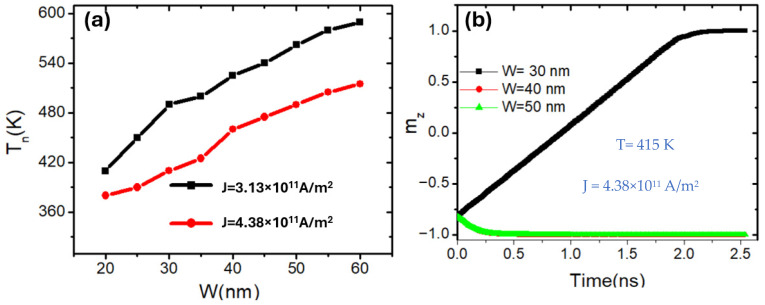
(**a**) Domain wall nucleation temperature *T_n_* as a function of nanowire width (*w*) under two different current densities: J = 3.13 × 10^11^ A/m^2^ (black squares) and J = 4.38 × 10^11^ A/m^2^ (red dots). (**b**) Time evolution of the normalized out-of-plane magnetization component m_z_ for three different widths (30 nm, 40 nm, and 50 nm), under device temperature of 300 K and J = 4.38 × 10^11^ A/m^2^. *K_u_* = 0.6 × 10^5^ J/m^3^ and *M_s_* = 750 kA/m. The nanowire dimensions were fixed at 200 nm length and 5 nm thickness.

The simulation was conducted to investigate the effects of nanowire geometry specifically width and thickness on the thermal behavior of domain wall (DW) nucleation and magnetization reversal in perpendicularly magnetized nanowires. In this part of the study, the nanowire width was varied between 20 nm and 60 nm to evaluate its influence on thermal switching and domain wall dynamics. Figure 3 presents two important insights into the thermal behavior of domain wall nucleation and magnetization reversal in perpendicularly magnetized nanowires. In subfigure (a), the domain wall nucleation temperature *T_n_* is plotted as a function of nanowire width (*w*) for two different current densities. A clear trend is observed; *T_n_* increases almost linearly with increasing wire width for both current densities. This is attributed to the enhanced total magnetic volume in wider nanowires, which increases the energy barrier against thermal nucleation. The increase of T_n_ with wire width can be understood from the geometry dependence of the total anisotropy and demagnetization energies. A wider nanowire possesses a larger magnetic volume, which enhances the effective perpendicular anisotropy energy contributing to the nucleation barrier. As a result, higher thermal energy is required to overcome this barrier, leading to an increase in Tn. In addition, the reduced demagnetizing field in wider geometries further stabilizes the uniform magnetization, delaying the onset of thermal nucleation.

The narrowest wire (30 nm) exhibits full magnetization reversal, while wider wires remain magnetically stable, indicating resistance to domain wall nucleation under the same thermal and current conditions. Moreover, the current density significantly affects the nucleation behavior. For a higher current density *J* = 4.38 × 10^11^ A/m^2^, the nucleation temperature is consistently lower than that of the lower current density case *J* = 3.13 × 10^11^ A/m^2^. This trend highlights the role of spin-transfer torque (STT), which becomes more effective at destabilizing the magnetization as current increases, thereby requiring less thermal energy to trigger domain wall nucleation. Subfigure (b) shows the temporal evolution of the normalized out-of-plane magnetization component *m_z_* for nanowires of different widths at a fixed elevated temperature. The results reveal a strong width dependence of the reversal dynamics. For the narrowest wire (30 nm), the magnetization undergoes a complete reversal within approximately 2 ns, signifying successful domain wall propagation and switching. In contrast, the wider wires (40 nm and 50 nm) maintain a stable *m_z_* close to −1.0 throughout the simulation, indicating that no domain wall nucleation or switching occurred. These results confirm that increasing the nanowire width reduces DW thermal switching, which in turn enhances the thermal stability of the domain wall under identical temperature and current conditions.

Together, these results establish a direct correlation between nanowire width, current density, and thermal activation of domain walls in PMA systems. The findings provide crucial design guidance for engineering thermally robust and controllable spintronic memory devices, especially in nanoscale geometries where thermal effects are pronounced. Compared to in-plane magnetized nanowires, PMA structures exhibit a fundamentally different thermal response. While domain wall nucleation in in-plane systems becomes easier with increasing width, PMA systems show enhanced thermal resistance due to the cumulative perpendicular anisotropy energy. Similarly, PMA reversal is more stable and less prone to spontaneous switching, making it highly favorable for thermally robust spintronic memory devices, even though the switching speed may be slower than that observed in in-plane geometries [44].

**Figure 4:**
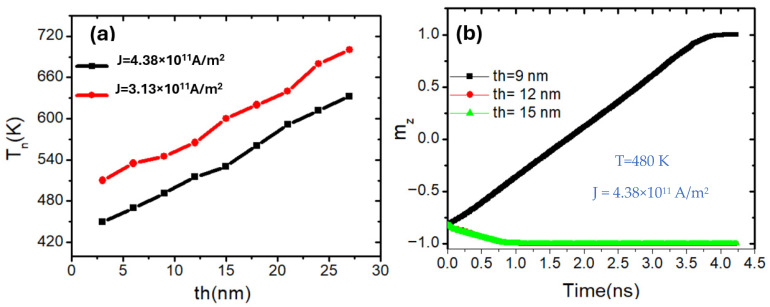
(**a**) Domain wall nucleation temperature (*T_n_*) as a function of nanowire thickness (*th*) for two current densities: J = 3.13 × 10^11^ A/m^2^ (red squares) and J = 4.38 × 10^11^ A/m^2^ (black dotes). The nucleation temperature increases with thickness, reflecting enhanced thermal stability due to the increase in total anisotropy energy. (**b**) Time evolution of the normalized out-of-plane magnetization component (*m_z_*) for three nanowire thicknesses (9 nm, 12 nm, and 15 nm). Only the 9 nm wire undergoes complete magnetization reversal within 4 ns, while thicker wires remain nearly stable at *m_z_* ≈ −1, demonstrating their higher resistance to thermally assisted switching. *K_u_* = 0.6 × 10^5^ J/m^3^ and *Ms* = 750 kA/m. The nanowire dimensions were fixed at 200 nm length and 40 nm width.

Another key geometric parameter that critically influences domain wall (DW) thermal behavior in perpendicularly magnetized nanowires (PMA) is the thickness of the device. Figure 4 provides critical insights into how the thickness of a perpendicularly magnetized nanowire affects its thermal response and magnetization dynamics. Subfigure (a) shows the domain wall nucleation temperature (*T_n_*) as a function of nanowire thickness (*th*) for two different current densities. A clear positive correlation is observed that *T_n_* increases steadily with increasing thickness for both current levels. This behavior stems from the fact that a thicker nanowire contains a larger magnetic volume (V), which directly contributes to a higher total anisotropy energy. Since the anisotropy energy scales with volume, thicker wires provide a larger energy barrier against thermal excitations, making domain wall nucleation increasingly difficult at a given temperature [13].

E = K_u_⋅V (2)

Furthermore, at any given thickness, a higher current density (black curve: *J* = 4.38 × 10^11^ A/m^2^) results in a lower nucleation temperature compared to a lower current density (red curve: *J* = 3.13 × 10^11^ A/m^2^). This is expected, as increased spin-transfer torque (STT) assists domain wall nucleation, effectively reducing the required thermal energy input. However, the relative impact of current density diminishes as thickness increases, indicating that the dominant factor becomes the thermal robustness imposed by the geometric scaling.

Subfigure (b) further reinforces these findings by showing the time evolution of the normalized out-of-plane magnetization component *m_z_* for three nanowires of varying thickness (9 nm, 12 nm, and 15 nm) under constant thermal and current conditions (480 K and J = 3.13 × 10^11^ A/m^2^). The thinnest wire (9 nm) exhibits complete magnetization reversal over approximately 4 ns, highlighting successful domain wall propagation. In contrast, the wires with thicknesses of 12 nm and 15 nm maintain a stable magnetization near m_z_ = −1.0, showing minimal response to the same thermal and current environment. This reinforces the idea that thicker wires exhibit stronger resistance to domain wall nucleation and propagation due to their higher anisotropy energy and magnetostatic self-energy.

From a physical standpoint, increasing the thickness of a PMA nanowire does not significantly alter the direction of the anisotropy (still perpendicular to the plane), but it amplifies the energy required for domain wall initiation. While this geometric robustness is highly desirable for memory retention and stability, it imposes a trade-off in energy efficiency and switching speed, as more current or thermal energy is needed to trigger switching in thicker structures [13].

The results for both width (Figure 3) and thickness (Figure 4) demonstrate a consistent trend: increasing either dimension of the nanowire enhances the thermal stability of the magnetization by raising the nucleation temperature. This behavior can be attributed to the increase in total magnetic volume, which scales the anisotropy energy barrier (Equation (2)) and thus requires more thermal energy to initiate domain wall formation. Although width and thickness were studied separately, their influence is physically equivalent within the investigated ranges. Together, these findings establish that larger magnetic volume whether achieved by increasing width or thickness, systematically suppresses thermally induced domain wall nucleation.

Having established the critical role of nanowire geometry, specifically width and thickness, in influencing the thermal stability of domain walls, it is equally important to explore how these geometric parameters affect the dynamic behavior of DWs under operational conditions. While wider and thicker nanowires exhibit enhanced resistance to thermally induced domain wall nucleation due to their larger magnetic volumes and higher anisotropy energy, these same features can significantly alter the velocity and response time of domain wall motion under applied current and temperature. Domain wall dynamics, including velocity and mobility, are key performance indicators in spintronic devices, as they determine the speed and reliability of information transport and switching [7]. Therefore, understanding how domain wall velocity evolves with temperature in geometrically defined nanowires provides deeper insight into the trade-offs between thermal robustness and switching performance. In the following part, we analyze domain wall dynamics in conventional PMA nanowires of 200 nm length, 40 nm width, and 5 nm thickness by examining how DW velocity responds to increasing temperature.

**Figure 5:**
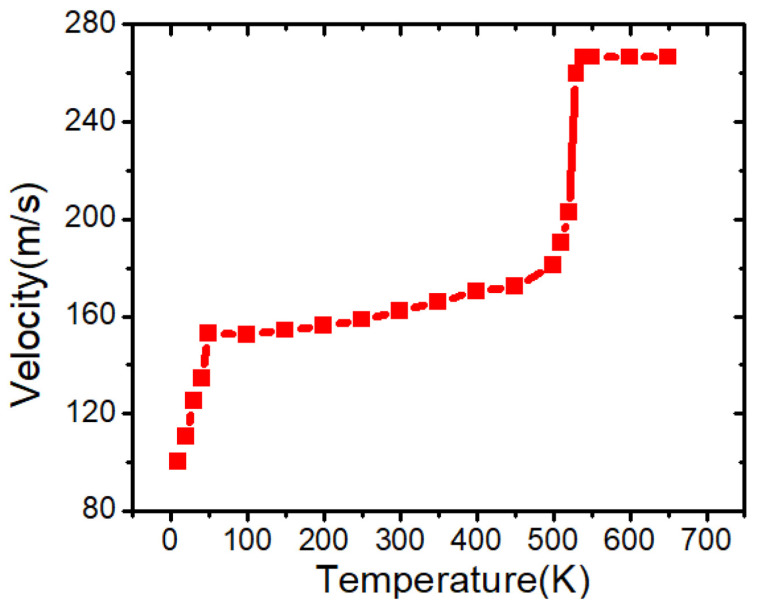
Domain wall velocity as a function of temperature in a magnetic nanowire with perpendicular magnetic anisotropy (PMA). The graph reveals three distinct regimes: a rapid increase in velocity at low temperatures (0–50 K) due to initial thermal activation, a gradual rise between 50 K and 500 K associated with thermally assisted DW motion, and a sharp transition to a high-velocity flow regime beyond 500 K. The velocity saturates at approximately 270 m/s. The nanowire dimensions were fixed at 200 nm length, 40 nm width, and 5 nm thickness. *K_u_* = 0.6 × 10^5^ J/m^3^ and *M_s_* = 750 kA/m. J = 3.13 × 10^11^ A/m^2^.

As shown in Figure 5, three distinct regimes of DW motion are identified with increasing temperature: (i) a creep regime at low temperatures, where velocity increases slowly due to pinning effects [18], (ii) a flow regime characterized by thermally activated motion and a stronger velocity increase [21], and (iii) a saturation behavior near the Curie temperature resembling the Walker plateau reported in PMA systems [13,38,42].

The graph in Figure 5 depicts the temperature dependence of domain wall (DW) velocity in a conventional PMA nanowire under constant current density of 3.13 × 10^11^ A/m^2^, revealing three distinct dynamical regimes. At low temperatures (0–50 K), the DW velocity increases rapidly from approximately 100 m/s to around 150 m/s. In this regime, the motion is thermally activated but still limited by intrinsic magnetic damping and the lack of sufficient thermal energy to assist spin dynamics. Even minor increases in temperature can significantly reduce effective damping, enabling faster domain wall propagation. The increase here is steep due to the initial suppression of thermal fluctuations at cryogenic temperatures.

From 50 K to ~500 K, the DW velocity plateaus and then gradually increases from about 150 m/s to 180 m/s. This behavior corresponds to a thermally assisted creep regime, where the domain wall is not pinned by geometric defects but still influenced by internal energy barriers from exchange and anisotropy. The magnetic PMA nanowire in this case is free of artificial pinning sites, so the DW motion is mostly governed by energy dissipation due to damping and the balance between spin-transfer torque (STT) and thermal agitation. The relatively flat behavior in this range highlights the fact that thermal energy increases the rate of DW hopping over internal energy barriers, but the overall velocity is still limited by the strength of anisotropy and the lack of sufficient driving torque.

A notable transition occurs between 500 K and 550 K, where the velocity experiences a sharp jump, reaching approximately 270 m/s. This behavior indicates the system’s entry into the flow regime, where the DW overcomes the thermal threshold necessary to maintain a continuous motion across the wire.

It should be noted that, in our simulations, the onset of the flow regime appears above 500 K. This value is system-dependent and reflects the particular choice of material parameters used in the model (e.g., *M_s_*, *K_u_*, and geometry). In experimental PMA systems, the transition into the flow regime is typically observed at significantly lower temperatures, often near room temperature, depending on the stack and current density [18]. Therefore, the exact temperature value in our simulations should not be interpreted literally, but rather as an indication of the general trend of thermally assisted flow dynamics.

At this point, thermal energy fully supports DW depinning and accelerates magnetization dynamics. The magnetic system effectively undergoes a reduction in anisotropy energy due to temperature softening of magnetic parameters (such as *M_s_* and *K_u_*), allowing the domain wall to respond more efficiently to the applied STT.

Beyond 550 K, the DW velocity saturates despite continued temperature increase. This saturation marks the dominance of damping and possibly thermally induced spin disorder, which limits further enhancement in speed. The observed plateau is characteristic of the Walker breakdown-free flow regime in PMA systems, where the domain wall maintains a stable Néel-type structure under thermal and current-driven forces without transitioning to oscillatory motion. This velocity saturation observed above 550 K can also be understood in relation to the Curie temperature (Tc) of the ferromagnetic system. As the temperature approaches Tc, both *M_s_* and *K_u_* decrease significantly, lowering the effective pinning barriers and initially enabling faster DW motion. However, near Tc, strong spin disorder and enhanced damping dominate, restricting further velocity increase despite the presence of higher thermal energy. However, in the present simulations both *M_s_* and *K_u_* were kept fixed and not varied with temperature. Therefore, this interpretation remains speculative, and the limitation is acknowledged here to distinguish between the simulation constraints and the actual physical behavior near Tc [42]. It should be noted that in the present simulations both *M_s_* and *K_u_* were kept fixed and not varied with temperature. Therefore, this indicates a potential research gap for further investigation, which may help to predict and explain the distinction between the simulation constraints and the actual physical behavior near Tc. This behavior is consistent with the critical slowing down of domain wall dynamics near the ferromagnetic paramagnetic transition, where increasing spin disorder and enhanced damping suppress the response of the wall despite higher thermal energy [41].

In contrast, in in-plane magnetized nanowires, the DW velocity often peaks and then decreases due to Walker breakdown. The absence of such instability in PMA systems is a significant advantage for memory technologies, as it ensures consistent and reliable switching performance over a broader temperature range. Equation (3) describes the thermally activated creep regime of domain wall motion, valid below the Walker breakdown field. This expression captures the exponential dependence of velocity on temperature and driving force [18], but does not account for the full velocity behavior across flow or Walker regimes

(3) $$v=v_{0}exp[-\frac{U_{c}}{k_{B}T}(\frac{f_{c}}{f}{)}^{\mu }]$$

where $v_{0}$ is a constant (prefactor), $U_{c}$ is the characteristic energy barrier, $k_{B}$ is the Boltzmann constant, T is the device temperature, f is the applied driving force (e.g., magnetic field or current) and μ is the creep exponent, typically taken as *μ* = 0.25 [19].

**Figure 6:**
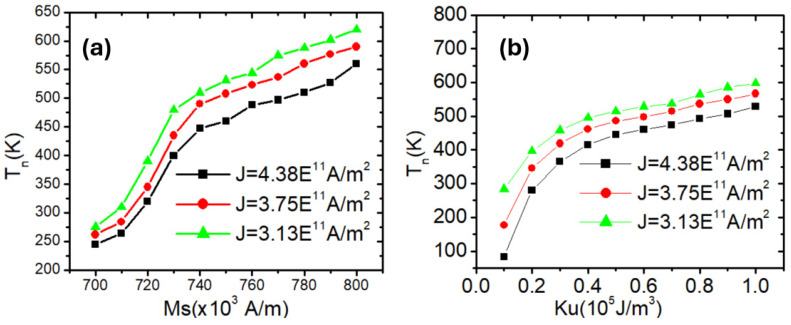
(**a**) Domain wall nucleation temperature (*T_n_*) as a function of saturation magnetization (*M_s_*) and (**b**) uniaxial anisotropy constant (*K_u_*) in perpendicularly magnetized nanowires under three different current densities: J = 3.13 × 10^11^ A/m^2^, 3.75 × 10^11^ A/m^2^, and 4.38 × 10^11^ A/m^2^. The nanowire dimensions were fixed at 200 nm length, 40 nm width, and 5 nm thickness.

In addition to geometric design, the magnetic properties of a nanowire play a fundamental role in determining its thermal and dynamic behavior, particularly in systems with perpendicular magnetic anisotropy (PMA). Material parameters such as the saturation magnetization (*M_s_*) and the uniaxial anisotropy constant (*K_u_*) directly influence the energy landscape that governs domain wall (DW) nucleation and motion. By tuning these properties, it is possible to engineer the energy barrier required for DW magnetization reversal, thereby tailoring the thermal stability and switching characteristics of the device. A higher *K_u_* enhances the out-of-plane anisotropy, increasing the energy needed for thermally induced DW nucleation, while a larger *M_s_* strengthens magnetic ordering and further resists thermal fluctuations. Understanding how these intrinsic magnetic properties affect the nucleation temperature is essential for optimizing the balance between data retention and switching efficiency in PMA-based spintronic applications. In the following analysis, we systematically investigate how variations in *M_s_* and *K_u_* influence the DW nucleation temperature under different current densities. Thus, in this study, the influence of magnetic properties on domain wall (DW) thermal stability and switching was investigated. In Figure 6a, the nucleation temperature increases monotonically with increasing *M_s_* for all three current densities. This is attributed to the fact that higher *M_s_* enhances the magnetic moment density, which increases the total Zeeman energy and contributes to a stronger magnetic ordering. As a result, more thermal energy is required to destabilize the magnetization and initiate a domain wall.

At lower current densities (e.g., 3.13 × 10^11^ A/m^2^, green triangles), the thermal stability is higher, evidenced by the consistently greater *T_n_* across all *M_s_* values. This indicates that the system resists domain wall nucleation more effectively when the spin-transfer torque (STT) influence is weaker. In contrast, higher current densities (e.g., 4.38 × 10^11^ A/m^2^, black squares) reduce *T_n_* by exerting stronger STT, which assists magnetization reversal and thus lowers the energy threshold needed for nucleation.

Figure 6b shows that *T_n_* exhibits a strong nonlinear dependence on the uniaxial anisotropy constant *K_u_*, particularly in the low-to-intermediate *K_u_* regime. This trend is expected because the energy barrier for magnetization reversal in PMA systems is directly proportional to *K_u_*⋅V, where V is the magnetic volume.

At low *K_u_*, even a modest increase in anisotropy leads to a substantial rise in Tn, as the system transitions from a thermally unstable to a stable regime. However, as *K_u_* exceeds 0.6 × 10^5^ J/m^3^, the curves begin to saturate, indicating that the DW nucleation barrier becomes very high and additional gains in stability require exponentially more thermal energy.

Similarly to the behavior with *M_s_*, higher current densities reduce *T_n_* due to the aiding effect of STT in overcoming the anisotropy barrier. The spacing between the curves becomes smaller at higher *K_u_*, suggesting that the relative influence of current density diminishes as the system becomes more anisotropy-dominated.

While the increase in current density J straightforwardly raises the nucleation temperature due to its proportionality with the spin-drift velocity u, the dependence on intrinsic material parameters such as *M_s_* and *K_u_* is more subtle. At low-to-moderate values, increasing *M_s_* or *K_u_* enhances the magnetic volume energy barrier, thereby significantly raising Tn. However, at higher studied values, the increase in Tn slows down. This saturation arises because thermal agitation becomes less effective once the anisotropy barrier and demagnetizing fields reach a critical balance. In particular, for large *M_s_*, the stronger dipolar interactions and exchange fields partially offset further gains in stability, while for large *K_u_*, domain wall width becomes very narrow, increasing wall energy but simultaneously reducing its mobility. As a result, the effect of further increasing *M_s_* or *K_u_* on *T_n_* diminishes, leading to the observed slowdown [46].

Experimentally, the simulation parameters used in this study such as variation in saturation magnetization and anisotropy can be realized by tuning the composition and thickness of multilayer PMA stacks (e.g., [Co/Pt], [Co/Ni], or [Ta/CoFeB/MgO]). Techniques such as magneto-optical Kerr effect (MOKE) microscopy, MFM, or spin-polarized scanning electron microscopy can be used to track domain wall nucleation and motion under thermal and electrical stimuli.

## 4. Conclusions

This study comprehensively explored the thermal stability and dynamic behavior of domain walls (DWs) in perpendicularly anisotropy magnetized nanowires (PMA), with a focus on how key magnetic parameters and geometric dimensions influence DW nucleation and motion. Using temperature-dependent micromagnetic simulations, we analyzed the influence of uniaxial anisotropy constant (*K_u_*), saturation magnetization (*M_s_*), nanowire width (*w*), and thickness (*th*) on the thermal nucleation temperature (*T_n_*) and DW velocity. Our findings reveal that the DW thermal nucleation behavior is highly sensitive to both material and structural properties. Specifically, increasing *K_u_* or *M_s_* significantly enhances the thermal stability of the DW, thereby raising the energy barrier against spontaneous domain wall formation. This effect is evident across various current densities, though higher current densities tend to lower *T_n_* due to the increased contribution of spin-transfer torque (STT), which assists in destabilizing the magnetic configuration. In terms of geometry, we demonstrated that both increased width and thickness lead to improved thermal resistance. Wider and thicker nanowires contain more magnetic volume, which results in a larger anisotropy energy, directly strengthening the stability of the magnetization against thermal excitation. However, this increase in thermal robustness introduces a trade-off; thicker and wider wires exhibit reduced responsiveness to applied current, slowing down domain wall propagation. Therefore, device design must consider this balance between thermal stability and switching speed to achieve optimal functionality. Dynamic simulations further showed that DW velocity increases with temperature, transitioning through three distinct regimes: (1) a rapid increase at cryogenic temperatures due to reduced damping, (2) a plateau region in the intermediate temperature range representing a thermally assisted creep regime, and (3) a sharp transition into a high-speed flow regime at elevated temperatures. Notably, PMA nanowires do not exhibit Walker breakdown, unlike in-plane magnetized systems, enabling more stable and predictable domain wall motion even under high thermal stress. These results emphasize the advantage of PMA systems for spintronic applications, particularly in racetrack memory and logic devices. The inherent thermal stability, combined with the absence of breakdown phenomena, offers a robust platform for data storage and processing at the nanoscale. By tailoring *K_u_*, *M_s_*, and geometric parameters, device engineers can strategically control the onset of DW nucleation and its velocity to meet specific application requirements. In conclusion, this work provides crucial insights into the temperature-dependent behavior of domain walls in PMA nanowires. The study offers a detailed phase-space of how thermal energy, material parameters, and structural factors interact to affect magnetic stability and dynamics. These insights pave the way for designing thermally resilient, energy-efficient spintronic memory devices, and highlight key parameters that can be optimized to strike a balance between data retention, switching reliability, and speed. Future research may build on these findings by integrating experimental validation and exploring more complex geometries or multilayered structures that further enhance control over thermal and dynamic behavior.

## Data Availability

Data is contained within the article.
